# Recombinant *Fasciola hepatica* fatty acid binding protein suppresses toll-like receptor stimulation in response to multiple bacterial ligands

**DOI:** 10.1038/s41598-017-05735-w

**Published:** 2017-07-14

**Authors:** Marcos J. Ramos-Benítez, Caleb Ruiz-Jiménez, Vasti Aguayo, Ana M. Espino

**Affiliations:** University of Puerto Rico, Medical Sciences Campus, Department of Microbiology, PO BOX 365067, San Juan, Puerto Rico 00936 USA

## Abstract

Recently, we reported that a native *Fasciola hepatica* fatty acid binding protein (FABP) termed Fh12 is a powerful anti-inflammatory protein capable of suppressing the LPS-induced expression of inflammatory markers *in vivo* and *in vitro*. Because the purification of a protein in native form is, in many situations not cost-beneficial and unsuitable for industrial grade scale-up, this study accomplished the task of optimizing the expression and purification of a recombinant form of FABP (Fh15). Additionally, we ascertained whether this molecule could exhibit a similar suppressive effect on TLR-stimulation and inflammatory cytokine expression from macrophages than those previously demonstrated for the native molecule. Results demonstrated that Fh15 suppresses the expression of IL-1β and TNFα in murine macrophages and THP1 Blue CD14 cells. Additionally, Fh15 suppress the LPS-induced TLR4 stimulation. This effect was not impaired by a thermal denaturing process or blocked by the presence of anti-Fh12 antibodies. Fh15 also suppressed the stimulation of various TLRs in response to whole bacteria extracts, suggesting that Fh15 could have a broad spectrum of action. These results support the possibility of using Fh15 as an excellent alternative for an anti-inflammatory drug in preclinical studies in the near future.

## Introduction

Fatty acid binding proteins (FABPs) in platyhelminths constitute a multigenic family of cytoplasmic proteins with isoforms localized in tegument and parenchymal cells. Parasitic trematodes are unable to synthesize lipids *de novo*, in particular long-chain fatty acids and cholesterol^1, 2^. Therefore, they use carriers to uptake such lipids directly from the host and transport them to specific destinations within parasite, a process in which FABP could play an important role. Thus, a major reason for interest in trematode FABPs relies in their potential role in drug delivery^3^ and the fact that *Fasciola hepatica* and *Schistosoma mansoni* FABPs may be cross-protective antigens^4–7^.

We recently reported that a native 12 kDa member of the *F*. *hepatica* FABP (Fh12) significantly suppresses the cytokine storm and other inflammatory mediators induced by lipopolysaccharide (LPS), which is the potent endotoxin of Gram-negative bacteria. In doing so, Fh12 functions as an antagonist of TLR4, a receptor that is targeted by the bacterial endotoxin and that is involved in the inflammatory response in cases of septicemia/septic shock^8^ and ulcerative colitis (UC)^9, 10^. When the mechanism of was studied, we found that Fh12 achieves its anti-inflammatory effect by targeting the CD14 co-receptor, which blocks the LPS-CD14 binding and stops the entire TLR4 signaling cascade from the beginning of the LPS-stimuli. Fh12 also activates the macrophages to an alternative pathway^11^, suppresses the microbial phagocytosis and suppresses the phosphorylation of various kinases downstream TLR4 (p38, ERK and JNK) that are common to multiple TLR-pathways^12^. Thus, during the infection, *F*. *hepatica* antigens, having FABP as a constituent could be saturating CD14 located on the surface of macrophages making them refractory to subsequent stimuli. Based on this particular mode of action, we considered that Fh12 is an attractive molecule with potential to develop a drug against sepsis, UC or any other inflammatory disease in which TLR4 is involved. A major limitation to exploit the anti-inflammatory potential of this molecule and perform pre-clinical studies is the difficulty to obtain large and homogenous batches of pure Fh12. The protocol optimized to purify Fh12 is long; the protein yield is relatively low and is therefore not cost-beneficial and unsuitable for scale-up at industrial level. A feasible alternative to solve this pitfall would be the production of stable clones expressing recombinant forms of Fh12 and the optimization of its expression using either a prokaryote or eukaryote expression system. However, Fh12 is considered a mix of isoforms of similar molecular masses (12 kDa) and different isoelectric points^13^ and we are unaware whether the anti-inflammatory effect showed by Fh12 could be mimicked by a recombinant variant of a single FABP isoform. Because *E*. *coli* is the first choice of host when a protein has to be expressed, the main goal of this study was to optimize the expression of a recombinant FABP termed Fh15 in *E*. *coli*, and determine whether Fh15 is able to mimic the anti-inflammatory properties showed by Fh12. Results demonstrated that Fh15 displayed a similar capacity than that of Fh12 to suppressed the expression of IL-1β and TNFα in murine macrophages and THP1 Blue CD14. Additionally, Fh15 also suppressed the LPS-induced TLR4 stimulation. Importantly, Fh15 also suppressed the stimulation of various TLRs in response to various whole bacteria extracts and this effect was not impaired by a thermal denaturing process or blocked by the presence of anti-Fh12 antibodies. Furthermore, data demonstrates that this recombinant version of FABP constitutes an excellent alternative in contrast to the purification of the native molecule, and it exerts a broader suppressive effect on the activation of various TLRs. These results support the possibility of testing Fh15 as an anti-inflammatory drug in preclinical studies in the near future.

## Results

### Production of recombinant Fh15

**Figure 1:**
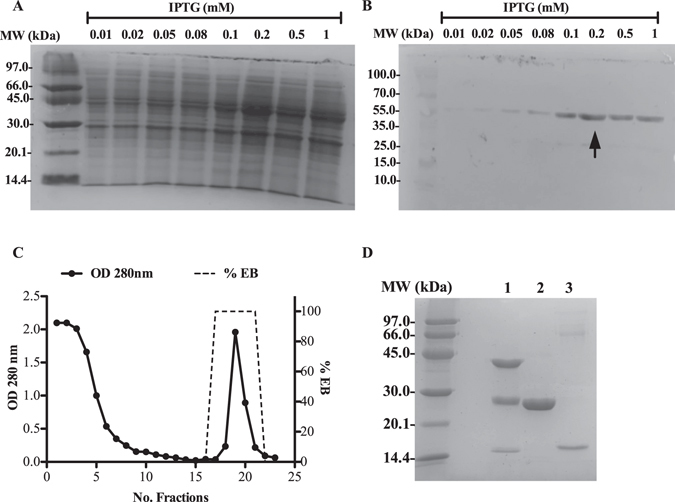
Optimization of the Fh15 expression in *E*. *coli* TOP10. cDNA encoding Fh15 was cloned into the pGEX-4T-2 and expressed in *E*. *coli* bacteria as a fusion protein with glutathione S-transferase (GST) of *Schistosoma japonicum* at the amino end of protein. Small-scale protein expression using 4-ml of LB medium was induced for 3 h at 27 °C, 225 rpm. (**A**) Bacteria lysates induced at various concentrations of IPTG were analyzed by 15% SDS-PAGE Coomassie blue stained. (**B**) Unstained gel was transferred to nitrocellulose membrane and incubated with specific anti-GST antibody labeled with peroxidase. Arrow indicates maximal expression of the GST-tagged protein estimated to be ~41–43 kDa, observed at 0.02 mM IPTG. (**C**) Fusion protein was purified from a large-scale expression culture using a GSTrap FF 5 ml column in an AKTA FPLC System. Fusion protein is eluted using the elution buffer (EB): 10 mM Tris-HCl pH 8.0 containing 10 mM GSH. (**D**) The purification process was analyzed by 15% SDS-PAGE. **Lane**-**1**: GST-Fh15 fusion protein, **lane**-**2**: GST-tag and **lane**-**3**: Fh15. Figure 1A,B and D represent cropped images from the original and are being displayed in black & white. Original full-length gels and blots are shown in Supplementary Figure 1.

The cDNA expressing Fh15 was cloned in the pGEX-4T-2 expression vector^14^ and the construct was propagated and expressed in *E*. *coli* TOP10. To determine the optimal conditions that render maximal Fh15 expression, eight different concentrations of IPTG were tested at different temperatures. Maximal expression of Fh15 was obtained with 0.2 mM IPTG at 27 °C (Fig. 1A,B). This temperature was low enough to prevent the formation of inclusion bodies^15^. At these conditions, the yield of Fh15 was ~3–4 mg per 1 liter of bacterial culture. Figure 1C,D shows the chromatogram and electrophoregram of the purified Fh15. A single protein band of ~14.7 kDa is always observed irrespectively of whether the electrophoresis is performed in reducing or non-reducing conditions, which confirms the absence of disulfide bonds in the protein moiety of this molecule (data not shown).

### Immunoreactivity and thermal stability of Fh15 and Fh12

**Figure 2:**
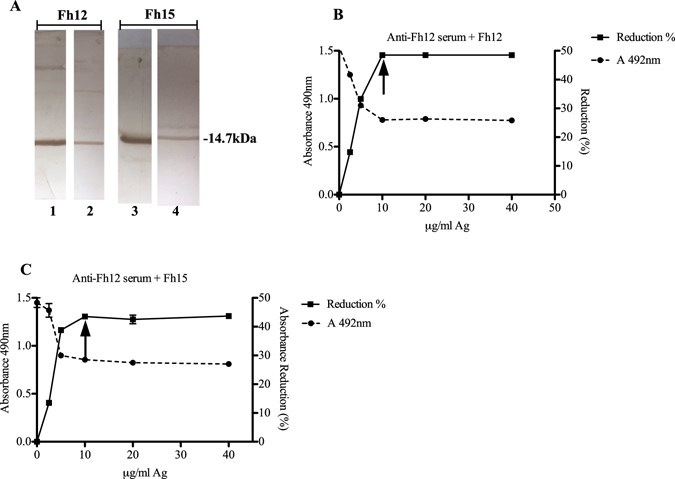
Western blot and Inhibition ELISA analysis of purified Fh15 with anti-Fh12 or anti-ESP serum. Immunological identity between Fh15 and Fh12 was investigated. (**A**) Fh15 and Fh12 were analyzed by 15% SDS-PAGE, electrotransferred to nitrocellulose membranes and incubated with anti-Fh12 antibody (lanes 1 & 3) revealing a strong immunoreactive band of ~14.7 kDa, or incubated with anti-ESP antibody (lanes 2 & 4), respectively, revealing the band of ~14.7 kDa at weaker intensity. Figure 2A represents cropped images from the original. Original full-length gels and blots are shown in Supplementary Figure 1. In the Inhibition ELISA, the plate was coated with 15 μg/ml Fh12. Anti-Fh12 antibody (diluted 1:200) was mixed with increased amounts of Fh12 (**B**) or Fh15 (**C**) ranging from 2.5 to 40 μg/ml and after 1 h incubation at 37 °C was added to the plate. The secondary antibody was further added diluted 1:5000 followed by the substrate solution. Maximal inhibition values of ~43.5–48.5% were obtained at concentrations starting at 10 μg/ml protein, which is indicated on the figure with an arrow.

Western blot analysis was used to determine whether Fh15 and Fh12 exhibit similar immunoreactivity against rabbit anti-Fh12 or anti-ESP serum. Both proteins (15 μg) were loaded onto a 15% SDS-polyacrylamide gel, run at similar conditions and then electrotransferred onto a nitrocellulose membrane and incubated with an anti-Fh12 or anti-ESP serum (diluted 1:400). The reaction was revealed by incubation with a goat anti-rabbit IgG peroxidase labeled and subsequent addition of substrate solution. A strong immunoreactive band of ~14.7 kDa was observed when the anti-Fh12 serum was tested against Fh12 or Fh15. Both antigens were also reactive with the anti-ESP serum showing a band of 14.7 kDa. However, this band was significantly weaker (Fig. 2A). The observation that both, Fh15 and Fh12 reacted weakly with the anti-ES serum indicates that fatty acid binding proteins could be minor components in the excretory-secretory products of *F*. *hepatica*.

To determine whether Fh15 shares immunological identity with Fh12, we applied an inhibition ELISA as previously described^16^. The ELISA plate was pre-coated with 15 μg/ml Fh12 and after blocking the anti-Fh12 serum (1:200) was added. Prior the addition to the plate, the anti-Fh12 serum had been pre-incubated for 1 h, 37 °C with different concentrations of Fh12 or Fh15 (ranging from 2.5 to 40 μg/ml) to favor the antigen-antibody complex formation. Thus, only free antibodies that are not forming immune-complexes with the antigen are available for binding to the coated antigen on the plate. After a washing step for eliminating the excess of reagents, the secondary antibody (goat anti-rabbit IgG-HRP) and the substrate solution were sequentially added as previously described^16^ and the absorbance at 490 nm (A_490_) was read after a 30 min incubation. Results showed an inverse correlation between the amount of antigen added to serum and the absorbance values. Maximal reductions of absorbance values were 43.5% (Fh15) and 48.5% (Fh12) when the proteins were added to the anti-Fh12 serum at a concentration of 10 μg/ml. Thus, both proteins showed similar inhibition curves suggesting a similar immunological identity between both molecules (Fig. 2B,C).

**Figure 3:**
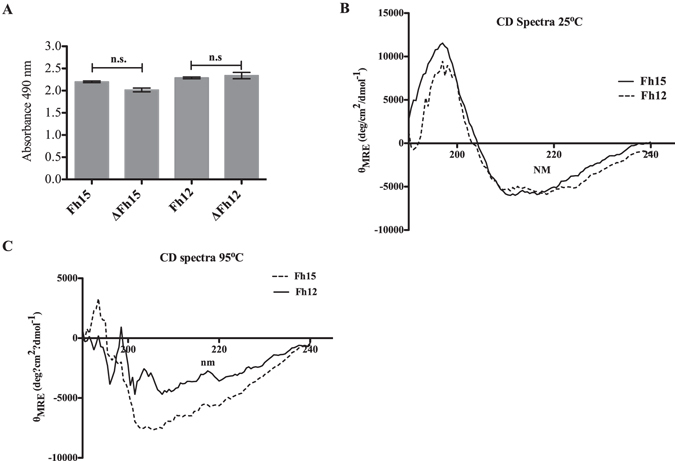
Immunoreactivity of Fh12 and Fh15 and Circular Dichroism spectra of Fh12 and Fh15 before and after a denaturing heat treatment. (**A**) Indirect ELISA was used to evaluate the immunoreactivity of Fh12 and Fh15 against the anti-Fh12 antibody diluted 1:200 in PBST before (Fh12 or Fh15) and after heat treatment (ΔFh12 or ΔFh15). No significant (n.s.) differences were found between the absorbance values of Fh12 and ΔFh12 or between Fh15 and ΔFh15. (**B**) CD spectra of Fh15 (solid line) and Fh12 (dashed line). Spectra was obtained at 25 °C using each protein at 0.1 mg/ml. (**C**) CD spectra of Fh15 and Fh12 after denaturing temperature treatment of 95 °C using each protein at 0.1 mg/ml.

In order to determine whether the immunoreactivity of Fh15 and Fh12 with the anti-Fh12 serum could be modified after a denaturing process, we heated both proteins at 95 °C for 10 min in a water-bath and measured their reactivity against the anti-Fh12 serum immediately. Results showed that the heat treatment does not alter the capacity of Fh12 or Fh15 to react with the anti-Fh12 serum (Fig. 3A). The CD spectra of Fh15 and Fh12 are quite similar in shape and intensity and are consistent with those previously reported for human FABP isoforms^17^. The maximum in the spectra for Fh12 and Fh15 is between 196 to 200 nm and the minimum was observed at 211 nm (Fig. 3B). Values of the molar ellipticity at 211 nm (*θ*_211_) for Fh12 and Fh15 are quite close (−5490.24 and −6001.44, respectively), which demonstrates that Fh12 and Fh15 exhibit a similar secondary structure. Data collected at 95 °C demonstrated a notable change in the intensity of ellipticity values of both spectra. Values at 196–200 nm become negative for both proteins, whereas the minimum values at 211 nm (*θ*_211_) increased slightly for Fh12 (−4130.39) and reduced for Fh15 (6482.51) (Fig. 3C). This demonstrates that the thermal treatment applied in effect, destabilizing the secondary structure of both proteins. Collectively, these results demonstrate that Fh12 and Fh15 proteins exhibit similar physic-chemical properties.

**Figure 4:**
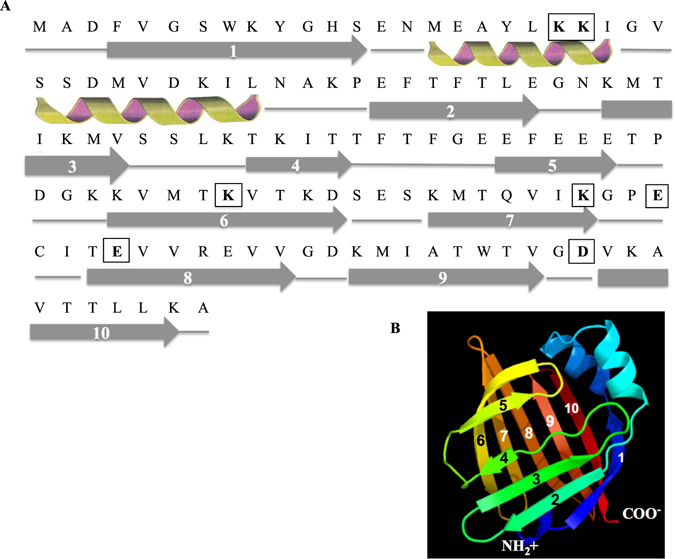
Prediction of secondary and tertiary structures of Fh15. (**A**) Secondary structures of Fh15 predicted by the SOPMA and (**B**) tertiary structure of Fh15 predicted by Phyre2. Both programs are available at ExPASy server (Bioinformatics Resources Portal, www.expasy.org). Fh15 contains two short alpha helixes (h) and a large content of extended strands (e) and random coils (c). Amino acid residues into boxes (K21, K22, K83, K97, E100, E104 and D122) are predicted to bind to the LPS-binding pocket on the human CD14 structure according previous studies^12^.

According to SOMPA secondary structure prediction software, the Fh15 structure is predicted to contain 12.0% of alpha helix, 59.0% of β-strand and ~29.0% of random coils (Fig. 4A). For Fh15, Phyre2 software predicted a tertiary structure consisting of 10 antiparallel β-strands that form a β-barrel, capped by two short α-helices arranged as a helix-turn-helix segment (Fig. 4B), which is consistent with the tertiary structure of FABP isoforms from other organisms^18–20^. A previous docking analysis revealed that Fh15 contains seven acid residues (K21, K22, K83, K97, E100, E104 and D122) that are predicted to interact with 8 amino acid residues in the LPS-binding pocket of the human CD14 protein moiety^12^. Based on the structural analysis performed in this study, only the residues K21 and K22 localize in the second short alpha helixes and the others residues localize in β-strands regions (Fig. 4B).

### Fh15 blocks the TLR-stimulation within THP1-Blue CD14 cells

**Figure 5:**
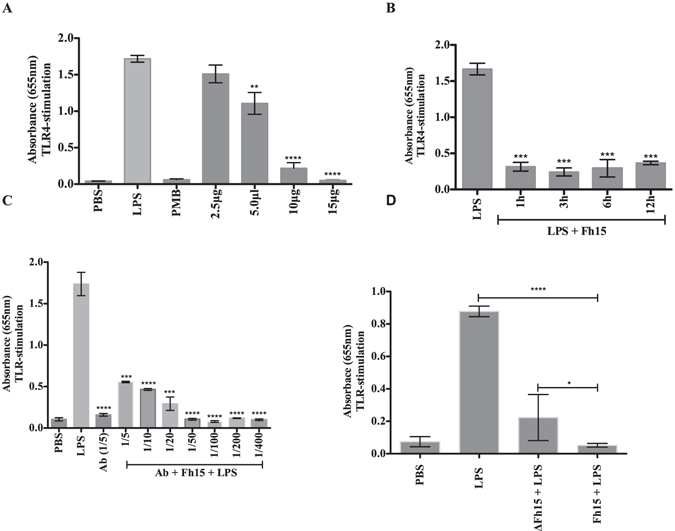
Effect of the heat treatment and the presence of anti-Fh12 antibodies on the capacity of Fh15 to suppress activation of different TLRs in THP1-Blue-CD14 cells. THP1 Blue-CD14 cells were seeded at 1 × 10^5^ cells/well in 96-well flat-bottom plates. The levels of SEAP secreted to the culture media were estimated by reading at 655 nm, 18 h after TLR-ligand stimulation. (**A**) Cells were cultured with Fh15 (2.5 to 15 μg/ml) before stimulation with LPS (1 μg/ml). Fh15 significantly suppressed the LPS-induced TLR4-stimulation at concentrations of 5 μg/ml (**p = 0.0012), 10 μg/ml (***p = 0.001) and 15 μg/ml (****p < 0.0001). (**B**) Cells were first stimulated with LPS (1 μg/ml) and then exposed to Fh15 (10 μg/ml) 1, 3, 6 and 12 h after LPS-stimulation. After 18 h of LPS stimulation, the QB-reagent is added to culture and readings at 655 nm were done 4 h later. Fh15 significantly suppressed the induced LPS-TLR4 stimulation at all time points tested after LPS-stimulation. ***p = 0.0001, **p = 0.001. (**C**) The anti-Fh12 serum (endotoxin-free) at dilutions ranging among 1:5 to 1:400 was added in triplicate to culture followed of the addition of Fh15 (10 μg/ml) before stimulation with LPS (1 μg/ml). Fh15 significantly suppressed TLR-stimulation induced by LPS and the presence of high anti-Fh12 antibody concentration (1:5) make no difference (***p = 0.0001, ****p < 0.0001). (**D**) Cells cultured with untreated or heat denatured Fh15 (ΔFh15) for 10 min at 95 °C before stimulation with LPS (1 μg/ml), demonstrate strong suppression against LPS-TLR4 induced stimulation (****p < 0.0001). Differences between both Fh15 and ΔFh15 proteins were also observed (*p = 0.015).

Having previously demonstrated that native Fh12 suppresses the stimulation of TLR4 and, consequently, the activation of the nuclear transcription factor NF-κB induced by LPS in HEK293-TLR4 cells^12^, we wanted to ascertain whether Fh15 could exert similar function. In the experiment, we used a human monocyte cell line (THP1-Blue CD14), which express multiple TLRs. These cells were cultured with different concentrations of Fh15 alone, or in the presence of various TLR-ligands at the concentration recommended by the manufacturer. By using dose-response analysis, optimal concentrations of LPS and the antagonist PMB were determined for subsequent analysis. A concentration of 1 μg/ml LPS induced maximal TLR4-stimulation, which was evidenced by the high levels of SEAP in the culture media indicative of activation of the NF-κB transcription factor. Concentrations of 100 μM PMB completely suppressed TLR4 stimulation and NF-κB activation. When Fh15 was added to cells, no stimulation was observed at any of the concentrations tested. However, when cells were cultured with Fh15 at concentrations of 10 or 15 μg/ml for 30 min prior stimulation with LPS, the levels of SEAP were significantly suppressed (*p* < 0.0001) by > 90%, which was similar to the effect of PMB control (Fig. 5A). Subsequent experiments were all performed with concentrations of Fh15 adjusted to 10 μg/ml since this was the lowest Fh15 concentration that rendered maximal suppression of TLR4-stimulation. To assess whether Fh15 could stop the TLR4-stimulation after the onset, we added 10 μg/ml Fh15 at different time points (1, 3, 6 and 12 h) after LPS-stimulation. Results demonstrate that the TLR4-stimulation induced by LPS was significantly reduced by 85.2% (*p* = 0.0002) when Fh15 was added to cell culture 1 h after LPS-stimulation. Reductions of 84.2%, 83.7% and 77.9% were also observed when Fh15 was added to cell culture 3, 6 and12h after LPS-stimulation respectively (*p* < 0.0001) (Fig. 5B). These results indicate that Fh15 suppresses the TLR4-stimulation induced by LPS is dose-dependent and that the timing of exposure makes no difference as was previously demonstrated in HEK293-TLR4 with the native Fh12^12^.

We wanted to further investigate whether in the presence of anti-Fh12 serum; the suppressive effect of Fh15 on the LPS-induced TLR4-stimulation could be abrogated. To address this hypothesis, we incubated THP1-Blue CD14 cells with different dilutions of the endotoxin-free anti-Fh12 serum, as was previously demonstrated with the chromogenic LAL assay (endotoxin levels < 0.1 EU/ml), and immediately thereafter, added Fh15 to culture followed by LPS-stimulation. Results showed that, even at the highest serum concentration (1:5 dilution), Fh15 significantly blocks (*p* = 0.0001) the LPS-induced TLR4-stimulation (Fig. 5C). Similar results were obtained irrespectively to whether Fh15 is added to culture at the same time, or prior to the anti-Fh12 antibody addition (data not shown). Moreover, the denatured protein (ΔFh15) reduced the levels of SEAP induced by LPS by 75%, which indicates that the thermal treatment applied to Fh15 does not affect its capacity to suppress the TLR4 stimulation induced by LPS (Fig. 5D). Values with untreated Fh15, however, were found to be significantly lower than those obtained with the ∆Fh15 (p = 0.015).

**Figure 6:**
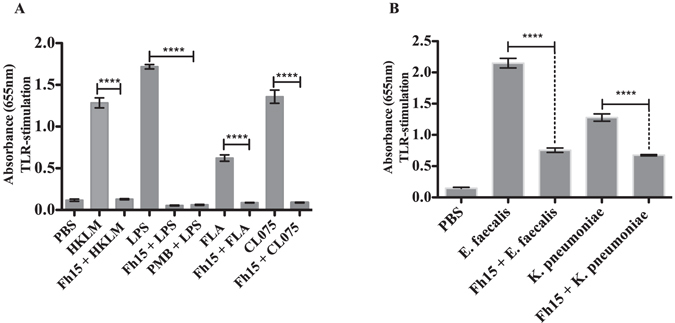
Fh15 suppresses the stimulation of multiple toll-like receptors. (**A**) THP1 Blue-CD14 cells were seeded at 1 × 10^5^ cells/well in 96-well flat-bottom plates and cultured with Fh15 (10 μg/ml) alone or 30 min prior to stimulation with optimized concentrations (established by the manufacturer) of heat killed *Listeria monocytogenes* (HKLM; 10^8^ cells/ml), flagellin (FLA; 1 μg/ml) or ortozialoquinoline (CL075; 10 μg/ml). Control cells were stimulated with PBS or a single TLR-ligand. (**B**) Cells were cultured with Fh15 (10 μg/ml) and then stimulated with whole extracts (1 × 10^8^ cells/ml) of *Enterococcus faecalis* or *Klebsiella pneumonia*. Cells incubated only with each bacteria extract were used as activation control. Fh15 significantly suppressed (****p < 0.0001) the stimulation induced by all TLR-ligands or whole bacteria extracts.

To determine whether Fh15 could also suppress the stimulation induced by other TLR-ligands, cells were incubated with 10 μg/ml of Fh15 for 30 min prior stimulation with HKLM (TLR2-ligand), FLA (TLR5-ligand) or CL075 (TLR8-ligand). Results demonstrated that Fh15 significantly suppressed the levels of SEAP in response to these ligands by 90.0%, 86.0% and 93.5%, respectively (Fig. 6A). This data suggests that, at the same experimental conditions, Fh15 could simultaneously suppress multiple TLRs and exhibit a broad spectrum of action. To confirm this assumption, cells were cultured with Fh15 (10 μg/ml) and 30 min later were stimulated with whole extracts of *K*. *pneumoniae* or *E*. *faecalis*, which are representative of Gram-negative and Gram-positive bacterial strains. Results demonstrated that Fh15 suppressed the stimulation induced by extracts of *K*. *pneumoniae* and of *E*. *faecalis* (Fig. 6B) by 52.0% (*p* < 0.0001) and 65.0% (*p* < 0.0001), respectively. These suppressions were very similar to the suppression caused by the PMB control, indicating that Fh15 suppresses the TLR2 or TLR4 stimulation induced by either Gram-negative or Gram-positive bacterial strains.

### Fh15 suppresses the expression of TNFα and IL-1β cytokines of murine bone marrow derived macrophages and THP1-Blue CD14 cells in response to LPS

**Figure 7:**
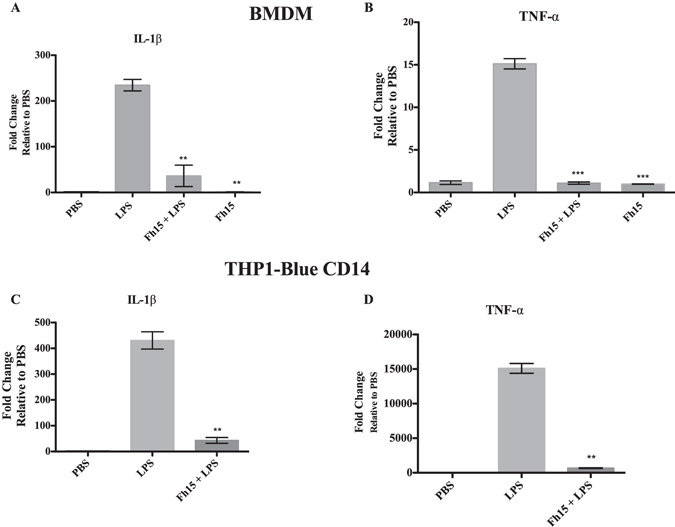
Fh15 suppressed the expression of TNFα and IL1β cytokines from mouse BMDM and human monocyte cell line (THP1-Blue CD14) in response to LPS. Bone marrow derived macrophages (BMDM) from naive C57BL/6 mice and THP1-Blue CD14 cells were treated with 5 μg/ml or 10 μg/ml Fh15, respectively 30 min before stimulation with LPS (100ng/ml or 1 μg/ml, respectively). Control cells were treated with LPS or PBS alone. After treatments cells were incubated by 18 h, at 37 °C 5% CO_2_. Expression of IL-1β and TNFα was measured by real-time RT-PCR. Results shown are expressed as fold-changes in expression relative to cells stimulated with PBS and presented as mean ± SD of a minimum of three experiments, each in triplicate. (**A** and **C**) Fh15 significantly suppressed the expression of IL-1β and TNFα and IL-1β in murine BMDM (**p < 0.01 and ***p < 0.001), respectively. (**B** and **D**) Fh15 also significantly suppressed the expression of IL-1β and TNFα in THP1 Blue-CD14 cells.

Having previously demonstrated that native Fh12 (5 μg) suppresses the expression of a large number of pro-inflammatory and inflammatory cytokines from murine BMDM^12^, we proceeded to determine whether recombinant Fh15 could have a similar function. We extracted cells from bone marrow, differentiated them into macrophages *in vitro* and then exposed cells to 10 μg Fh15 prior to stimulation with LPS. The expression of TNFα and IL-1β was measured by real-time RT-PCR 24 h after LPS-stimulation. As expected, cells stimulated with LPS overexpressed high levels of TNFα or IL-1β, which are a signature of an ongoing inflammatory response^21, 22^. In contrast, cells failed to express TNFα or IL-1β cytokines both, when they were exposed to Fh15 alone and in the mixture of Fh15 + LPS (Fig. 7A,B). Suppression of TNFα or IL-1β was found to be highly significant (*p* < 0.001). Similar results were obtained when TNFα and IL1β expression from THP1 Blue CD14 cells treated with LPS or the Fh15 + LPS mixtures were analyzed (*p* < 0.0012) (Fig. 7C,D). These results indicate that Fh15 down regulates the expression of inflammatory cytokines at similar intensity to that previously demonstrated by native Fh12^12^.

**Figure 8:**
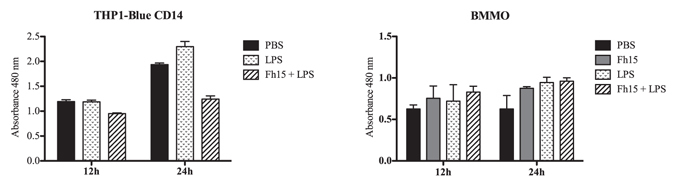
Cell viability. Mouse BMDM and THP1-Blue CD14 cells were exposed to Fh15, LPS or Fh15 + LPS during 12 or 24 h. Cell viability was determined by adding 50 μl XTT to each well. After an additional incubation of 24 h at 37 °C the absorbance of each well was read at 480 nm.

To rule out that the suppressive effect showed by Fh15 on THP1-Blue CD14 cells or BMDM is due to toxicity, we measured the influence of Fh15 on cell viability using an XTT assay. The results demonstrated that the treatment of THP1-Blue-CD14 cells or BMDM with Fh15, LPS or the Fh15 + LPS mixture for 12 or 24 h did not compromise the cell viability (Fig. 8A,B).

## Discussion

*F*. *hepatica*, is a ‘master of immunomodulation’ as are many other parasitic helminths. This parasite polarizes the immune system of the mammalian host into a dominant Th2 status with significant suppression of the Th1-inflammatory responses^23–26^. As a result, hosts infected with *F*. *hepatica* are rendered much more susceptible to secondary bystander infections, such as *Bordetella pertussis* and *Mycobacterium tuberculosis*, which require Th1 immunity for protection^25–27^. Such a polarizing effect is mediated by a milliard of molecules secreted by the parasite that are termed excretory-secretory products (ESPs), which can mimic the immunomodulatory effect that is observed during active infection with *F*. *hepatica*, without the tissue pathology^23, 24^.

Our research group was the first to report the anti-inflammatory capacities of a 12 kDa fatty acid binding protein (Fh12), a property that had been only documented so far for parasite ESPs^23, 26, 28^ or tegument extracts^29, 30^. Although fatty acid binding proteins (FABPs) are cytosolic proteins that lack peptide-signals in their protein moiety, many proteomic analyses have identified FABP in the *F*. *hepatica* ESPs and in the tegument^31–33^ indicating that these proteins are in contact with the host immune system during the active infection. In fact, a recent proteomic study identified *F*. *hepatica* FABP in the parasite secretome^34, 35^, suggesting that this molecule can be secreted from the parasite into exosome-like vesicles.

Our previous study demonstrated that Fh12 activates macrophages into a non-inflammatory phenotype through TLR4 and, in doing so, functions as an LPS antagonist blocking the inflammatory mediators induced by LPS *in vivo* (in a murine model of septic shock)^12^ and *in vitro*, both in murine macrophages, and in human monocytes^11, 12^. Based on these antecedents, we considered Fh12, a promising molecule for the development of anti-inflammatory drugs. Considering that purification of a protein in its native form is always a hard task that in many situations is not-cost effective and unsuitable for industrial level scale-up, the rationale behind this study was to optimize the expression and purification of a recombinant variant of *F*. *hepatica* FABP in *E*. *coli*. We will also want to ascertain whether it could exhibit a similar suppressive effect on TLR-stimulation and inflammatory cytokine expression from macrophages than those previously demonstrated for the native molecule (Fh12).

Because Fh15 is the best biochemically characterized recombinant variant of *F*. *hepatica* FABPs and its sequence is available in the GenBank, we selected this protein as the target of our study. Fh15 was isolated for the first time by Rodriguez-Perez *et al*.^14^ by screening a cDNA library from *F*. *hepatica* adult flukes using an anti-polyclonal antibody raised against a 12 kDa *F*. *hepatica* antigen purified in native form from a crude *F*. *hepatica* extract using ion exchange chromatography^36^. Fh15 is an acidic protein (estimated Ip = 6.0) with a predicted molecular weight of 14.7 kDa. It was designated Fh15 due to its similar molecular weight to a 14.8 kDa polypeptide (Sm14) of *Schistosoma mansoni*, another FABP of a platyhelminth reported^37^. Both, Fh12 and Fh15 proteins have been extensively used to induce protection to challenge with *F*. *hepatica* infection in mice, sheep^5, 38^ or rabbits^4^. Moreover, some studies have used Fh12 or Fh15 proteins to induce cross-protection against *Schistosoma mansoni* or *S*. *bovis* ^6, 7^. Despite Fh12 and Fh15 being theoretically different in molecular weight, the scientific community has largely considered them the same molecule. The differences in migration of both proteins in SDS-PAGE are almost imperceptible, as confirmed by results of this study (Fig. 2A), which are consistent with the observations of by other authors. The migration of Fh12 in SDS-PAGE toward the 14.7 kDa polypeptide has been attributed to possible post-translational modifications in the adult fluke^14^ or the overall effect of several FABPs isoforms from which Fh15 is only one of^16, 39^. The similarity of the CD spectra between Fh15 and Fh12 confirm that they exhibit the same secondary structure. Thus, although the exact amino acid residues of Fh12 protein remain unknown, the data subject that Fh12 possesses the same predicted tertiary structure, as does Fh15. In addition to the structural similarity, Fh12 and Fh15 also share immunological characteristics as demonstrated in the inhibition ELISA experiments and by the fact that even after a denaturing heat treatment they did not lose their capacity to react with the anti-Fh12 serum or suppress the TLR4-stimulation induced by LPS. The observation that even after denaturation Fh15 retained its capacity to suppress the TLR4-estimulation suggests that the tertiary and secondary structure of this protein may not be essential for its capacity to suppress the inflammatory response. This is in agreement with the fact that most of amino-acid residues presumably involved in the Fh15-CD14 binding interaction^12^ are localized in the extended strand regions. This finding suggests that perhaps, chimeric or synthetic variants of Fh15 containing these specific residues at a proper distance on the structure could also be as effective as recombinant Fh15 suppressing the inflammatory response. However, further studies are needed to confirm this assumption. The observation that the extreme temperature conditions did not disrupt the functional stability of Fh15 is of great importance. During manufacturing and distribution of pharmaceutical products, temperature excursion could potentially deteriorate the product quality^40^. Therefore, if in the near future Fh15 is formulated as a therapeutic anti-inflammatory option, data showed herein about the thermal stability of the Fh15-function could be important for the development of scaling up protocols, transportation and storage management.

Although Fh15 functions in a similar manner to Fh12, in order to maximize the suppressive effect on TLR-stimulation within THP1-Blue CD14 cells, or the expression of inflammatory cytokines within murine BMDM, it was necessary to employ a two-fold increase of Fh15 (10 μg) compared to Fh12 (5 μg). Differences in the amount of protein needed could be attributed to any of the following reasons. First, Fh12 is a mix of at least 6 acidic and slightly basic isoforms^16^ each one acting at the same time with similar and redundant mechanisms of action, whereas Fh15 is a single molecule and therefore, much more concentration is required to compensate the lack of other isoforms. Second, Fh12 is naturally expressed by the parasite, which is a eukaryote organism whose machinery to perform post-translational modifications is intact. In contrast, Fh15 is expressed in a prokaryote expressing system that is unable to undergo post-translational modifications because bacterial systems lack the organelles required for such modifications^41–43^. Third, Fh15 could also be expressed with a different folding, which in someway could affect its interaction with the CD14 co-receptor and consequently, more protein amount must be required to overcome this deficiency. Our analysis using the DictyOGlyc server^44^ to predict GlcNAc O-glycosylation sites in the Fh15 primary sequence demonstrated that Fh15 is not a glycosylated protein, which is consistent with the fact that they bind and transport lipids that are essentially hydrophobic^45^. Moreover, Fh15 was expressed in *E*. *coli* at low temperature conditions (27 °C), which increases the stability and correct folding patterns due to the fact that hydrophobic interactions determining inclusion body formation are temperature dependent^46^. Thus, based on these arguments, we reasoned that the first reason exposed above would be the most feasible explanation to the differences in concentration between Fh15 and Fh12 required to maximize the TLR-suppression observed in this study.

In addition, other interesting observations revealed in this study are that Fh15 was able to suppress the TLR4-stimulation up o 12 after LPS-stimulation in THP1 Blue CD14. This result suggests that Fh15 could be useful, not only as prophylactic drug to prevent the dissemination of the infection in the host, but also as treatment after the sepsis onset. Moreover, Fh15 also dramatically suppresses the TLR-stimulation induced by Gram-positive or Gram-negative bacterial extracts in these cells. *Enterococcus faecalis* is a Gram-positive commensal bacterium inhabiting the gastrointestinal tracts of humans and other mammals. *Klebsiella pneumoniae* is an opportunistic Gram-negative bacterium that commonly causes infections of urinary and respiratory tract^47^. Both species are frequently associated with nosocomial infections causing life threatening infections in humans^47^ and the high levels of antibiotic resistance found in *E*. *faecalis* and *K*. *pneumoniae* contribute to their pathogenicity^47^. The wall components of Gram-positive bacterium are recognized by TLR2, whereas bacterial cell components of Gram-negative bacterium, specifically LPS, are recognized by TLR4. However, although TLR2 and TLR4 have differential roles in recognition, both groups both are activated by bacterial components via the CD14 co-receptor^48^. CD14 has been also involved in the activation of TLR6^49^, and it is known to constitutively interact with the MyD88-dependent TLR7 and TLR9^50^. CD14 also plays a role in the stimulation of TLRs by viruses^50^. Knowing that one of the mechanisms used by *F*. *hepatica* FABP to achieve its anti-inflammatory role is binding to CD14^12^, and based on the observation that Fh15 was able to suppress the TLR-stimulation induced by bacterial extracts of *E*. *faecalis* and *K*. *pneumonia*, which express multiple molecular patterns that are simultaneously recognized by multiple TLRs, we concluded that Fh15 could exert a broad spectrum of action. The capacity of Fh15 to suppress the inflammatory response in response either to Gram-negative or Gram-positive bacteria strains could undoubtedly have relevant therapeutic applications in the near future.

The ability of Fh15 to suppress multiple TLRs in response to stimulation with bacterial ligands suggests this molecule could be an ideal therapeutic candidate for the treatment of chronic inflammatory conditions as sepsis/septic shock. Indeed, sepsis is not a common event during helminth infection^51^, which supports the therapeutic potential of helminth antigens. Using *F*. *hepatica* molecules to target specific innate cell signaling will circumvent the problem of global immune suppression associated with parasite infection and also with current immune-therapies. Synthetic anti-inflammatory drugs (Eritoran and Tak-242) have been successfully tested in experimental model of endotoxic shock^52^. The present study offers a recombinant, well-characterized *F*. *hepatica* protein (Fh15) with anti-inflammatory properties as a promising drug alternative. *In vivo* studies directed to corroborate this important function of Fh15 are being scheduled.

## Methods

### Ethics Statement

All experiments and procedures performed with animals or tissues collected from animals (inbred female or male C57BL/6 mice 6–8 week-old) were performed in accordance with relevant guidelines and regulations of the Institutional Animal Care and Use Committee of University of Puerto Rico, Medical Sciences Campus and approved by this committee (Protocol Number: 7870215).

### Whole bacteria extract preparation

Whole bacteria extracts (WBE) were prepared from heat attenuated, normal and multidrug resistant bacterial strains (*Enterococcus faecalis* and *Klebsiella pneumoniae*). Bacteria were cultured in Luria Bertani broth (LB) for approximately 8 hours and then were killed by boiling for 15 min in a water bath.

### *F*. *hepatica* ESPs, Fh12 purification and antiserum production

*F*. *hepatica* excretory-secretory products (ESPs) were obtained by *in vitro* maintenance technique of adult flukes freshly collected from infected livers at a local slaughterhouse as previously described^53^. Endotoxin-free native *F*. *hepatica* fatty acid binding protein (Fh12) was purified from an adult worm extract using a previously optimized protocol that involves sequentially an ultracentrifugation step at 30,000 × *g*, gel filtration chromatography and two preparative isoelectric focusing (IEF) steps as previously described^11^. Polyclonal antibodies against ESPs and Fh12 were produced in rabbits by subcutaneous injections of 200 μg of protein mixed with an equal amount of complete Freund’s adjuvant in the first injection, and incomplete Freund’s adjuvant in the boost injections as previously described^12^. Anti-sera had antibody titers of ~1:102,400 or 1:51,200 when they were titrated by ELISA against the Fh12 or ESPs, respectively.

### High-throughput protein expression and purification of recombinant *F*. *hepatica* fatty acid binding protein (Fh15)

cDNA encoding full length fatty acid binding protein from *F*. *hepatica* (Fh15) (GenBank: M95291.1)^14^ was cloned into pGEX-4T-2 vector as previously reported^6^. Clones expressing Fh15 as fusion protein with *Schistosoma japonicum* glutathione-S-transferase sequence (GST) were propagated and expressed in *Escherichia coli* BL21 bacteria. To optimize the conditions that favor maximal expression of GST-Fh15 fusion protein, small-scale protein expression in 4-ml LB medium with 100 μg/ml ampicillin were performed. Protein expression was induced at OD_600_ = 0.7 by addition of isopropyl β-tiogalactopyranoside (IPTG) at concentrations ranging among 0.05 to 1 mM at 27 °C during 3 h. The cultures were centrifuged at 4,000 rpm for 10 min at 4 °C and then resuspended in 1-ml cold lysis buffer (0.1 M phosphate buffered saline pH 7.4 (PBS) containing 1% Triton-X-100) and then subjected to two successive cycles of freeze/thaw. Lysates were then centrifuged at 10,000 g for 30 min, 4 °C. The supernatants were collected and analyzed by 15% SDS-PAGE stained with coomassie blue and Western blot in the presence of a specific anti-GST antibody labeled with peroxidase (anti-GST-HP) as described below.

### Scaled-up expression and purification of Fh15

Culture of *E*. *coli* transformants containing cDNA expressing GST-Fh15 fusion protein was scaled-up to 300-ml at 37 °C. Protein expression was induced at OD_600_ = 0.7 with 0.2 mM IPTG at 27 °C for 3 h. The recombinant GST-Fh15 protein was purified in a single step by using affinity chromatography with a HP5/5 GSTrap column in an AKTA FPLC system with Unicorn software. Absorbance of eluates was monitored at 280 nm. Total cell lysates were prepared from 300-ml culture in 20 ml of lysis buffer and loaded onto column at low flow rate (~1 ml/min). Unbound proteins were washed out with PBS. GST-tagged protein was eluted from column by washing with 10 mM Tris-HCL pH 8.0 buffer containing 10 mM reduced glutathione (GSH). GST-tagged protein was desalted against PBS using a PD-10 column (sephadex G-25). Recombinant Fh15 was excised from the GST-tag by incubation with Thrombin at a rate of 50 units thrombin by mg of fusion protein during 3 h at 4 °C with gentle agitation. After incubation, the digestion mix was reloaded onto the GSTrap column re-equilibrated with PBS and Fh15 was eluted by washing with PBS, whereas GST was eluted with 10 mM GSH as described above. The CNDB enzymatic assay was used for monitoring the efficiency of the GST-tagged protein binding to the column and recovery during the whole purification process.

### Endotoxin removal

Endotoxins were removed from Fh15 by using polymyxin B (PMB) columns according to the manufacturer’s instructions. The presence of endotoxins was assessed before and after removing endotoxins using the Chromogenic Limulus Amebocyte Lysate QCL-1000 Assay (Lonza, Walkersville, MD) following the manufacturer’s instructions. Endotoxin levels were quantified using a standard curve and reported as endotoxin units per milliliter. Protein concentration was adjusted to 1 mg/ml as determined by BCA method using a Pierce protein assay kit (Pierce, Cambridge, NJ). Purified endotoxin-free Fh15 was stored in aliquots at −20 °C until use.

### Secondary structure prediction, thermal stability and circular dichroism measurements

Secondary structure predictions of Fh15 were made using the SOPMA and Phyre2^54^ servers available at ExPASy (Bioinformatics Resource Portal, www.expasy.org). Fifteen micrograms of Fh15 or Fh12 in 50 mM phosphate buffer, pH 8.0 were heated by 10 min at 95 °C in a water-bath and immediately tested by ELISA against the anti-Fh12 serum. Absorption spectra were recorded spectrophotometrically at 20 °C with a scan speed of 20 nm/min (200–320 nm) and compared with those obtained before protein heat treatment. Alternatively, circular dichroism (CD) measurements in the far-UV region (190–350 nm) were performed with a Jasco J-1500 CD spectrometer and protein concentrations of 0.1 mg mL^−1^ in 50 mM phosphate buffer, pH 8.0 in the 190–250 nm ranges at 20 °C and 95 °C using 0.1-cm pathlength cell.

### Western blot analysis

Ten-micrograms of protein were analyzed on 15% SDS-PAGE and stained with coomassie-blue or electrotransferred to 0.45-μm nitrocellulose membranes (Bio Rad) as previously described^16^. The membranes were first blocked with 5% nonfat dry milk in PBS containing Tween-20 (PBST), then incubated 2 h at room temperature (RT) with anti-GST antibody labeled with horseradish peroxidase (HR) diluted 1:5,000 (GE Healthcare Life Sciences, USA) or incubated overnight with the anti-Fh12 or anti-ESP serum diluted 1:400 in PBST. After 3 washes with PBST, the membrane that had been incubated with the anti-GST-HP antibody was incubated at RT with the substrate solution (50-mg diaminobenzidine + 100 μl H_2_O_2_ 30% [w/v] + 100 ml PBS) until bands were visible, whereas the other membranes were incubated with the secondary antibody (goat anti-rabbit IgG-HP conjugated) diluted 1:5,000 in PBST. After another wash step, membranes were further incubated at RT with the substrate solution until bands were visible. To stop the reaction, membranes were soaked in distilled water.

### Indirect ELISA and Inhibition ELISA

Indirect ELISA was performed in disposable 96-well polystyrene plate (Costar, Corning New York), which was coated overnight with 15 μg/ml Fh15 (before or shortly after heat treatment) in 0.05 M carbonate buffer pH 9.6 as determined by checkerboard titration. After coating, the plate was washed 3 times with PBST and unbound sites in the wells were blocked with 3% non-fat dry milk diluted in PBST. After incubation for 1 h at 37 °C, the plate was emptied by suction and anti-Fh12 or anti-ESP serum diluted 1:200 in PBST was added to wells (100 μl/well) in duplicate and incubated for 1 h at 37 °C after which plate was washed 3 times. Conjugate (100 μl/well peroxidase-labeled goat anti-rabbit IgG) diluted 1:5,000 in PBST-milk was added and incubated 1 h at 37 °C and then washed again 3 times. Substrate (100 μl/well of 20 μl H_2_O_2_, 30% [wt/v] + 50 ml 0.1 M citrate buffer pH 5.0 + 20-mg *o*-phenylenediamine hydrochloride) was added and incubated in the dark at room temperature for 30 min, and the reaction was stopped with 50 μl per well of 10% HCl. Absorbance was read at 490 nm using a microplate ELISA reader (Bio Rad). In the inhibition ELISA, the anti-Fh12 serum was mixed with amounts of Fh15 or Fh12 ranging between 2.5 to 40 μg/ml and incubated 1 h at 37 °C to favor antigen-antibody complex formation. Further, the antigen-serum mix was added to plate and incubated 1 h at 37 °C and the protocol continued as described above.

### Screening assay using THP1-Blue-CD14 transfected cells

To investigate the effect of Fh15 on the activation of multiple TLR-pathways, we used THP1-Blue-CD14 cells, a cell line derived from human monocytes that stably express a macrophage-specific differentiation antigen (CD14) that interact with several TLRs. THP1-Blue-CD14 cells express and respond to ligands for TLR2, TLR4, TLR5 and TLR8 and are transfected with a reporter gene, secreted embryonic alkaline phosphatase (SEAP), driven by the NF-κB promoter (Invivogen, San Diego, CA). Upon TLR stimulation, cells activate the transcription factor and subsequently secrete SEAP, which is detected when the QUANTI-Blue^TM^ (QB) medium is added to the culture, which turns purple in the presence of SEAP. Cells were seeded in 96-well flat-bottom plates at 1 × 10^5^ cells/well in 100 μl of RPMI supplemented with 10% heat-inactivated fetal bovine serum fetal bovine serum (FBS), 50 U/ml penicillin, 50 μg/ml streptomycin, and 2 mM L-glutamine. In the stimulation experiments cells were treated with Fh15 (2.5 to 15 μg/ml) or WBE (1 × 10^8^ cells/ml) and incubated at 37 °C, 5% CO_2_ for 24 h. Cells treated with TLR-agonist at the concentration recommended by manufacturer (Invivogen, USA) were used as activation control. The TLR-agonists used in the study were lipopolysaccharide (LPS) (1 μg/ml), heat-killed *Listeria monocytogenes* (HKLM) (10^8^ cells/ml), flagellin (FLA) (1 μg/ml) and orthiazoloquinoline (CL075) (10 μg/ml). In the inhibition experiments, cells were cultured with Fh12 with or without heat treatment 30 min prior HKLM, LPS, FLA, CL075 or WBE stimulation. In other experiments, cells were first stimulated with LPS (1 μg/ml) for 1, 3, 6 or 12 h, after which Fh15 (10 μg) was added to the culture and incubated by additional 12 h. Afterwards, 20 μl of supernatant of each well was transferred to a clean 96-well flat-bottom plate, the QB reagent was added (180 μl/well) and the incubation prolonged for 5 h. Readings were done at 655 nm (A_655_). Cells treated with PBS were used as negative control and cells incubated with polymyxin-B (PMB) (100 μM) or Chloroquin (100 μM) were used as antagonist controls. To quantify the inhibition percentage in the levels of SEAP secreted to culture media produced by Fh15, we used the formula R (%) = 1 − [(A − C)/(B − C)] × 100, where A is the mean A_655_ of three replicates obtained when cells were cultured with Fh15, and B is the mean A_655_ value obtained when cells were exposed to TLR-ligands, and C is the mean A_655_ of three replicates obtained when cells were stimulated with PBS.

### TLR4 stimulation in the presence of anti-Fh12 antibody

To ascertain whether the anti-Fh12 antibody could neutralize the effect of Fh15 or Fh12, THP1-Blue CD14 cells were cultured with the anti-Fh12 serum at dilutions among 1:5 to 1:400 in endotoxin-free water and 5 min later were incubated with Fh15 or Fh12 (10 μg/ml) followed by the stimulation with LPS (1 μg/ml). Cells were then incubated at 37 °C, 5% CO_2_ for 18 h and then incubated with the QB-reagent for 5 h and absorbance were read at 655 nm as described above. As positive control, we used cells cultured with the antibody and stimulated with LPS and as negative control cells cultured only with the antibody, with PBS, Fh15 or Fh12.

### Isolation and treatment of mouse bone marrow–derived macrophages

BMDM were collected from femoral and tibial shafts of mice by flushing with 3 ml cold sterile PBS. The cell suspensions were passed through a sieve to remove large clumps, washed three times with sterile complete DMEM (supplemented with 20 mM L-glutamine, 1 ml penicillin and streptomycin/ 100 ml medium, and 10% heat-inactivated FCS; Sigma-Aldrich). Cells were adjusted to 1 × 10^6^ cells/well with differentiation medium (complete RPMI 1640 supplemented with 20 ng/ml M-CSF; R&D Systems) and cultured in 24-well plates (Nunc) at 37 °C, 5% CO_2_. On day 3 of culture, non-adherent cells were removed and the adherent cells were placed in fresh differentiation medium, and the incubation was prolonged for 7 days to cause full maturation of macrophages, which was assessed by FACS analysis and F4/80 surface Ag expression. BMDM were seeded into 24-well plates (Nunc) at 10^6^/ml in complete DMEM and then treated with 10 μg Fh15 for 30 min before being exposed to LPS (100ng/ml). Control cells were treated with PBS, Fh15, or LPS alone.

### Cell viability

To determine whether the optimized Fh15 concentration affects cell viability, THP1-Blue CD14 cells and BMDM were seeded at 1 × 10^6^ cells/well in 96-well flat bottom plates and treated with LPS (1 μg/ml), Fh15 (10 μg/ml) + LPS (1 μg/ml) for 12 h or 24 h at 37 °C. Following incubation, cell viability was assessed by adding 50 μl XTT (sodium 3′-[1-(phenylaminocarbonyl)-3,4 tetrazolium]-bis(4-methoxy-6-nitro) benzene sulfonic acid hydrate) labeling reagent (Roche Life Science, USA) to each well. The absorbance of each well was read at 480 nm.

### Quantitative real-time RT-PCR (qPCR)

Total RNA was extracted using a RNA isolation kit (Qiagen) kit, followed by treatment with Turbo DNA free endonuclease (Ambion, Grand Island, NY) to remove contaminating genomic DNA. RNA was quantified using a Nanodrop-1000 spectrophotometer (Thermo-Scientific, USA) and a high-capacity RNA-to-cDNA kit (Applied Biosystems, Carlsbad, CA) to perform the reverse transcription. cDNA was amplified using a StepOne Plus Real-Time PCR system (Applied Biosystems). cDNA was equivalent to 5ng total RNA and SYBR green PCR master Mix (Applied Biosystems). The cycling conditions were as follows: 95 °C for 15 min followed by 40 cycles of 95 °C for 15 s, 55 °C for 30 s, and 72 °C for 30 s. The primers used for each gene are listed in Table 1. Primer concentration was optimized and dissociation curves were generated for each primer set to verify the amplification of a single PCR product. qPCR experiments were conducted in triplicate using a StepOne Plus real-time PCR system (Applied Biosystems). The 2^−ΔΔCt^ method^55^ was used to quantify relative gene expression using GAPDH as an internal control and expressed as fold change relative to expression in the cells stimulated with PBS. The values reported are the mean of three replicates. The SD of the mean is shown as error bars in each group.

**Table 1:** Primers used in the qPCR experiments.

Murine Primers	Sequence (5′ to 3′)
IL-1β	Sense: GATCCACACTCTCCAGCTGCA
	Anti-sense: CAACCAACAAGTGATATTCTCCATG
TNFα	Sense: CATCTTCTCAAAATTCGAGTGACAA
	Anti-sense: TGGGAGTAGACAAGGTACAACCC
GPDH	Sense: AAGGTCATCCCAGAGCTGAA
	Anti-sense: CTGCTTCACCACCTTCTTGA
**Human Primers**	**Sequence** (**5′ to 3′**)
IL-1β	Sense: GCTCGCCAGTGAAATGATGG
	Anti-sense: GTCCTGGAAGGAGCACTTCAT
TNFα	Sense: TGGGATCATTGCCCTGTTGAG
	Anti-sense: TCTAAGCTTGGGTTCCGACC
β-Actin	Sense: ACAGAGCCTCGCCTTTGCCGAT
	Anti-sense: TTGCACATGCCGGAGCCGTT

### Statistical analysis

All data were analyzed for normality prior to statistical testing. When comparisons of the values for multiple groups were made, data were analyzed using one-way analysis of variance. For comparison of values for two groups, the Student’s *t*-test was used using Graphpad Prism software (Prism-6). For all tests, a *p* value of < 0.05 was deemed significant.

### Methods Statement

Authors declare that all experiments and procedures performed with animals or tissues collected from animals were performed in accordance with relevant guidelines and regulations of the Institutional Animal Care and Use Committee of University of Puerto Rico, Medical Sciences Campus and approved by this committee (Protocol Number: 7870215).

## Electronic supplementary material

Supplementary information.
