# Supplementary material for: Recombinant Fasciola hepatica fatty acid binding protein suppresses toll-like receptor stimulation in response to multiple bacterial ligands
Source: Sci Rep. 2017 Jul 14;7:5455. doi: 10.1038/s41598-017-05735-w (PMC5511235; doi:10.1038/s41598-017-05735-w)
Supplement: Supplementary file 1 — Supplementary information. [file 41598_2017_5735_MOESM1_ESM.pdf]

Supplementary Figure

Title: Recombinant *Fasciola hepatica* fatty acid binding protein suppresses toll-like receptor stimulation in response to multiple bacterial ligands.

Marcos J. Ramos-Benítez[1+], Caleb Ruiz-Jiménez[1+], Vasti Aguayo[1], and Ana M. Espino[1*]

[1] University of Puerto Rico
Medical Sciences Campus
Department of Microbiology
PO BOX 365067
San Juan, PR 00936
[*]Corresponding author: ana.espino1@upr.edu

Original 15% SDS-PAGE Coomassie Blue stained and Western blot analysis to optimize the IPTG concentration that induce maximal Fh15 expression (Figures 1A-B)

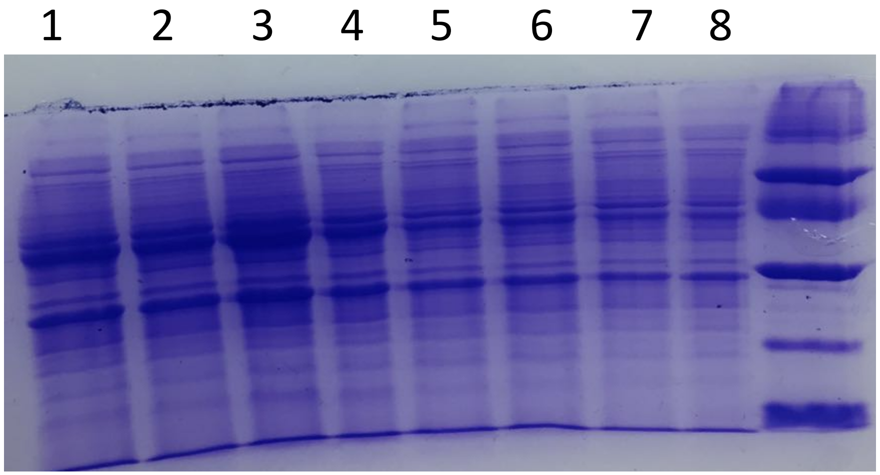

1: 1M IPTG
2: 0.5mM IPTG
3: 0.2mM IPTG
4: 0.1mM IPTG
5: 0.08mM IPTG
6: 0.05mM IPTG
7: 0.02mM IPTG
8: 0.01mM IPTG

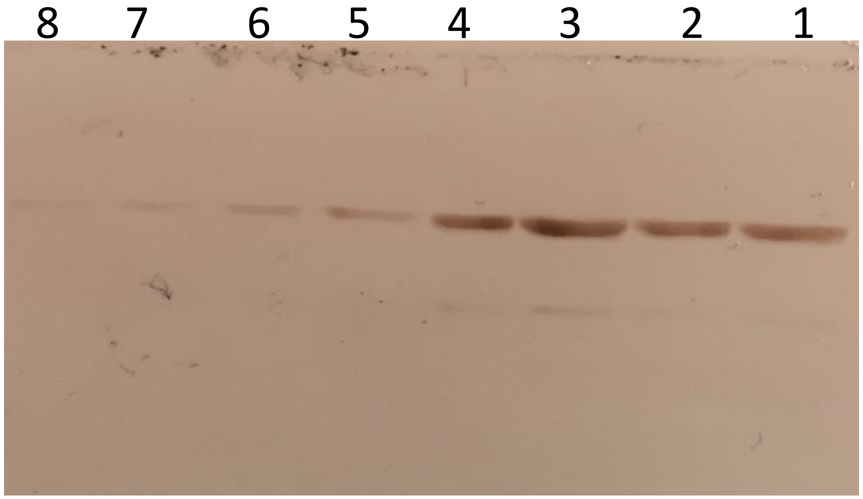

Primary Antibody:

Anti-GST-HRP from GE Healthcare Life Sciences USA

Original 15% SDS-PAGE Coomassie blue stained showing the analysis of GST-Fh15 Thrombin digestion (Fig. 1D)

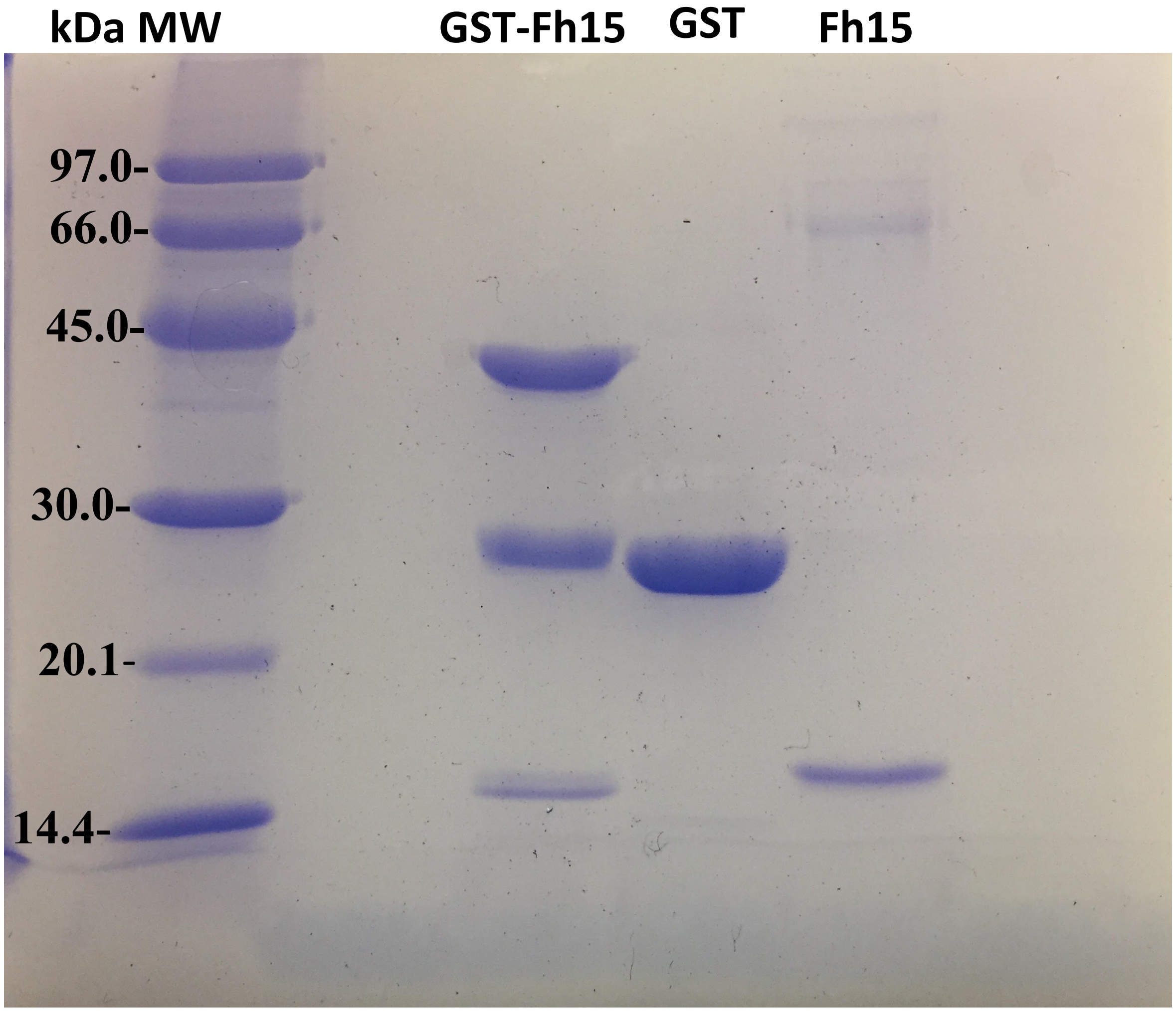

Original Blots showing the immunoreactivity of Fh12 and Fh15 against the anti-FABP and anti-ES serum, respectively.

Fh12

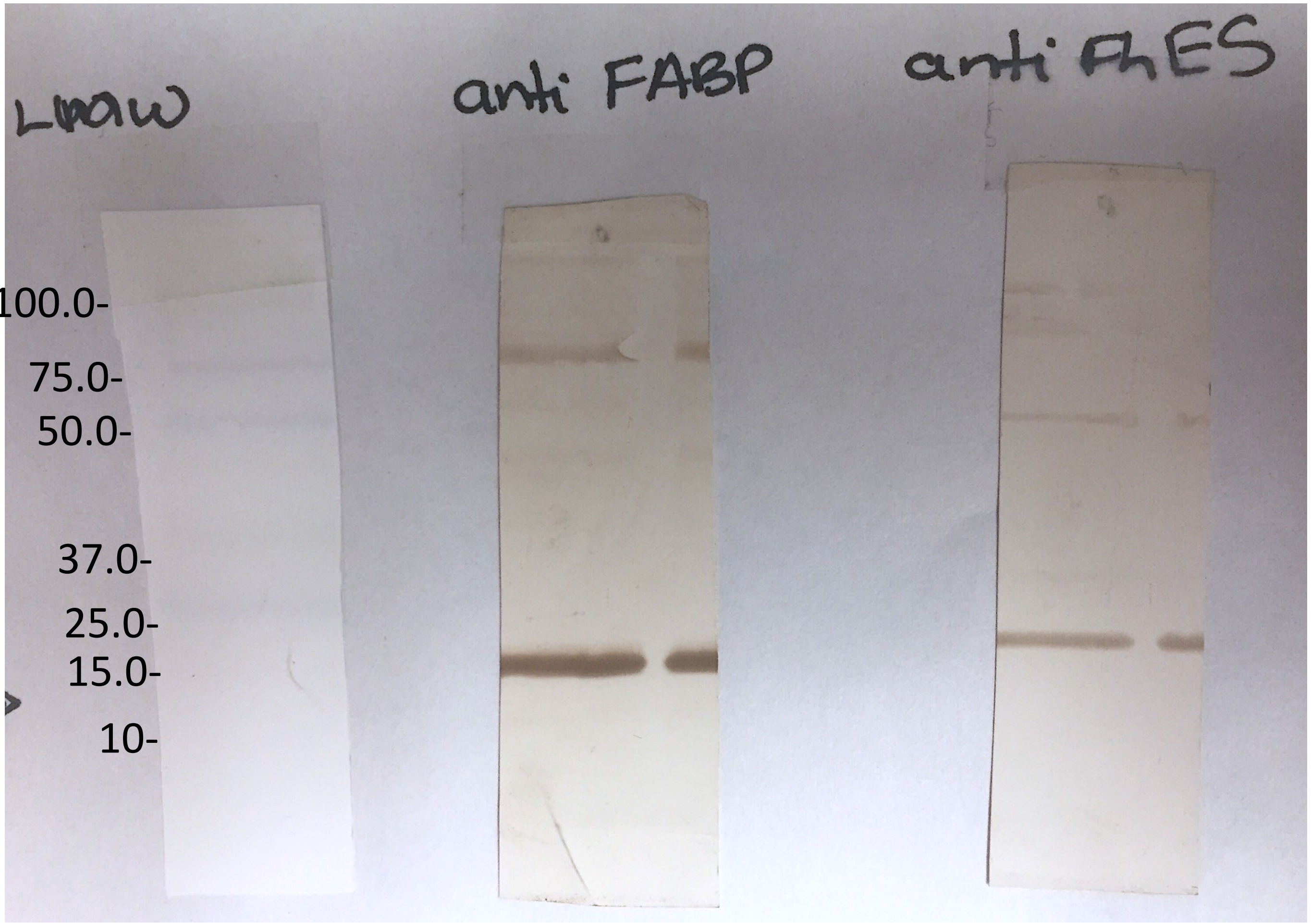

Fh15

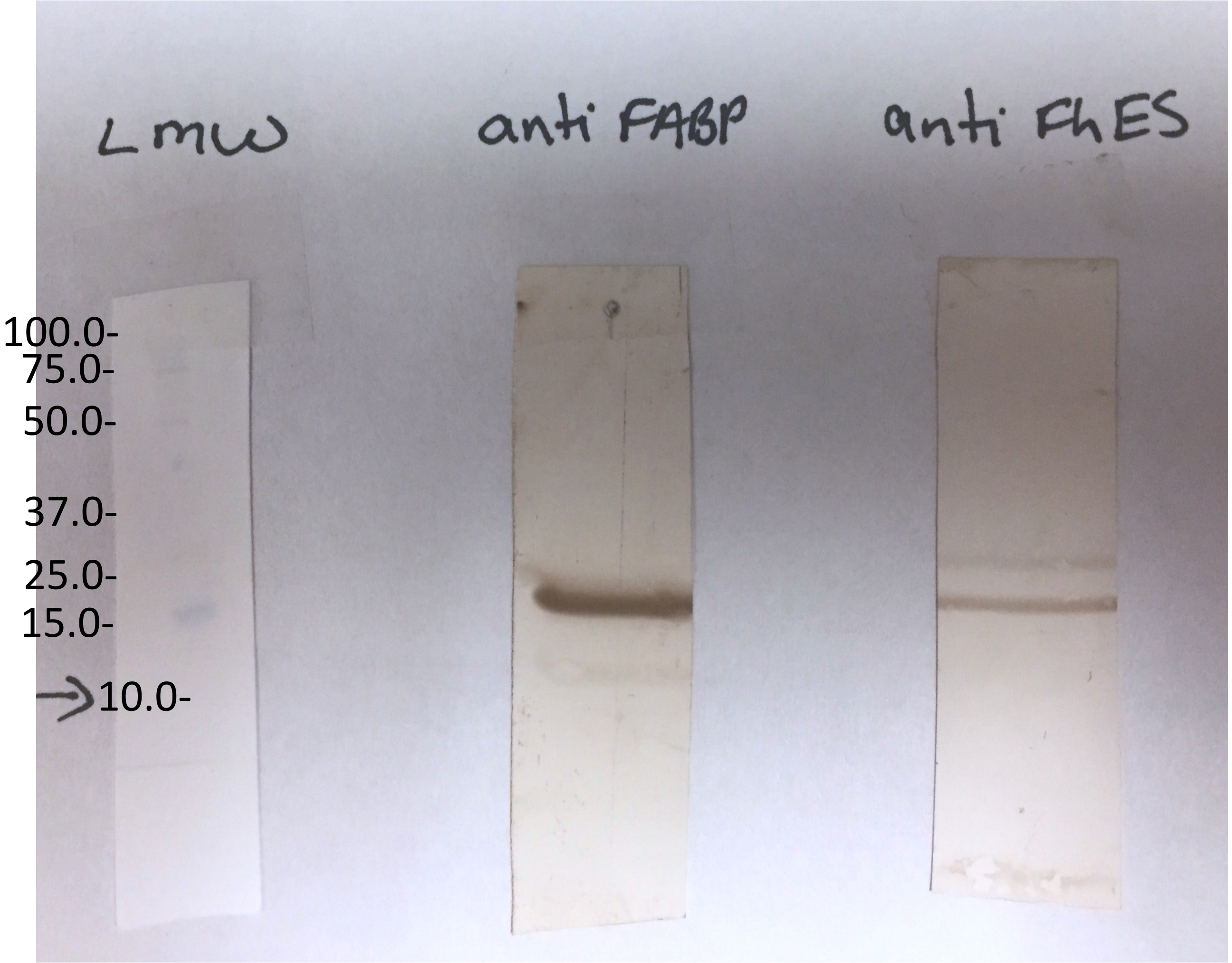

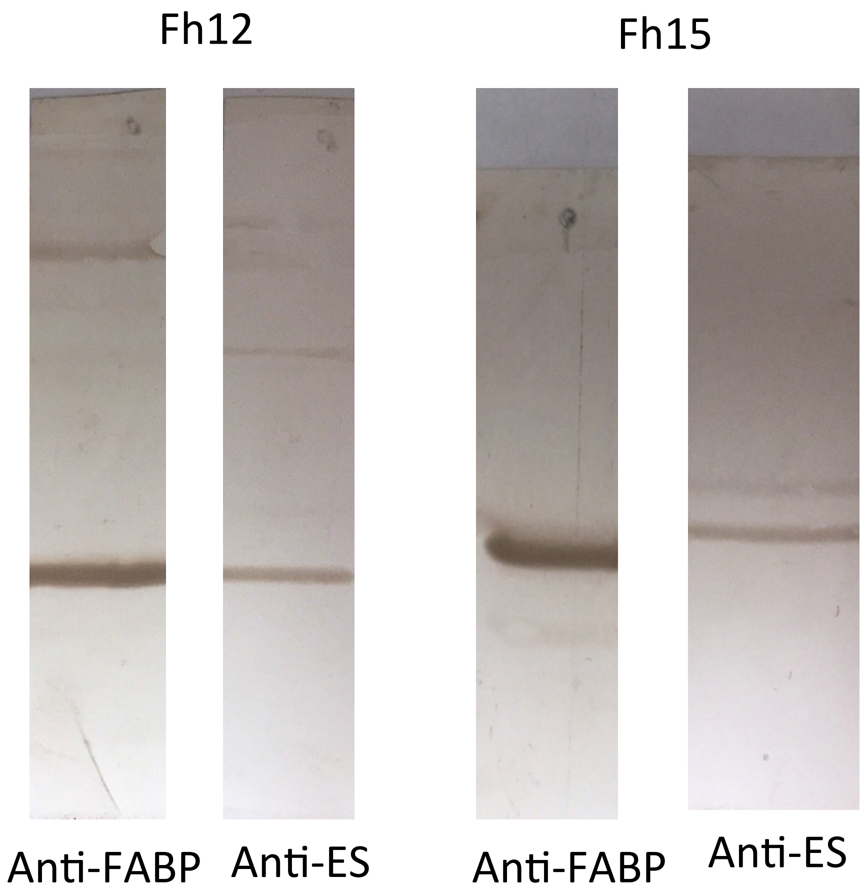

Anti-FABP serum: Polyclonal antibody obtained in rabbits by SC immunization with native Fh12 in Complete or Incomplete Freund Adjuvant

Anti-ES serum: Polyclonal antibody obtained in rabbits by SC immunization with FhESPs in Complete or Incomplete Freund Adjuvant
